# Endoscopic balloon dilation of esophageal stricture in dystrophic epidermolysis bullosa patient: challenges faced and safety of procedure

**DOI:** 10.1093/omcr/omae079

**Published:** 2024-07-30

**Authors:** Ruchi Mishra, Shivangi Tetarbe, Vinit Vinod Bedekar, Ira Shah

**Affiliations:** Department of Pediatric Gastroenterology and Hepatology, Bai Jerbai Wadia Hospital for Children, Acharya Donde Marg, Mumbai, 400012, Maharashtra, India; Department of Pediatric Gastroenterology and Hepatology, Bai Jerbai Wadia Hospital for Children, Acharya Donde Marg, Mumbai, 400012, Maharashtra, India; Department of Anesthesiology, Bai Jerbai Wadia Hospital for Children, Acharya Donde Marg, Mumbai, 400012, Maharashtra, India; Department of Pediatric Gastroenterology and Hepatology, Bai Jerbai Wadia Hospital for Children, Acharya Donde Marg, Mumbai, 400012, Maharashtra, India

**Keywords:** CRE balloon, total intravenous anesthesia, triamcinolone acetate, stricture dilatation

## Abstract

Background: Epidermolysis Bullosa (EB) stands as the prototype category of disorders featuring subepidermal fragility, characterized by skin blistering induced by minimal trauma. The gastrointestinal tract is a common site of extracutaneous injury. Esophageal stricture (ES) is one of the severe complications, with nearly 70% of patients experiencing ES within the initial 25 years of life. Case Report: We present a 11-year-old female child of dystrophic EB (DEB) who presented with dysphagia. Barium swallow showed a short segment proximal ES. We faced many challenges before endoscopy owing to difficult intravenous access, restricted mouth opening, multiple dental caries and low haemoglobin. Dental extraction under general anaesthesia and fibreoptic intubation with a smaller sized endotracheal tube guided over epidural catheter was done at another tertiary care institute. Child had severe bleeding due to airway manipulation. Management: At our centre endoscopy guided serial balloon dilation (BD) of ES was performed without intubation under total intravenous anaesthesia (TIVA) without any complications. The stricture was serially dilated under direct visualization till 12 mm in three sessions at three-weekly intervals using CRE (controlled radial expansion) fixed and wire-guided balloon dilators. During the first session 20 mg of triamcinolone acetate injection was also topically applied without mucosal invasion. No such further topical or submucosal applications were attempted due to risk of perforation. Conclusion: Endoscopy guided BD of ES is safe and effective in EB patients when done by experienced team.

## Introduction

Epidermolysis Bullosa (EB) stands as the prototype category of disorders featuring subepidermal fragility, characterized by blistering of skin induced by minimal mechanical trauma and disruption occurring at the dermo-epidermal junction. The four primary classical types of EB are EB simplex (EBS), junctional EB (JEB), dystrophic EB (DEB), and Kindler EB (KEB) [1]. The clinical spectrum of EB encompasses localized to widespread skin lesions, often accompanied by extensive multisystem involvement beyond the cutaneous domain [1].

In inherited EB, the gastrointestinal (GI) tract is a common site of extracutaneous injury with reports suggesting that two-third patients across all EB types suffer from some or the other GI symptom [2]. The GI complaints in EB patients arise because of formation of painful blisters and erosions anywhere in the GI tract from mouth to anus [3]. Severe and chronic luminal blistering may lead to scarring and the development of strictures [3]. A study of large series of patients has estimated cumulative risk of developing esophageal stenosis or stricture in DEB-HS patients (Hallopeau-Siemens), as per earlier classification, as 6.73%, 35.19%, 57.40%, 79.14%, and 94.72% by age 1, 5, 10, 20, and 45 years respectively [3]. Over the last two decades, there is a general consensus among clinicians that balloon dilatation is the preferred treatment for esophageal stricture in EB patients. We present a child of DEB with esophageal stricture who underwent endoscopic stricture dilatation.

## Case description

An eleven-year-old girl presented to our pediatric gastro-enterology out-patient department in January 2023 with dysphagia for three years. She was suffering from recessive dystrophic epidermolysis bullosa. At the onset of the complaint, family perceived the difficulty in swallowing solids could be due to pain from the trauma to oral mucosa on chewing solids, hence, the child preferred semi-solid food instead of solid food. Because the dysphagia progressed over the past three to four months, rendering the child unable to consume semi-solid food and consume only liquid food items [Dysphagia score-3 (Mellow-Pinkas scoring)], the family consulted the doctor. There was no history of any fever or cough or respiratory distress. The child had been born at full term, with a weight of 3 Kg, to a consanguineously married couple. Antenatal period was uneventful. The mode of delivery was caesarean section in view of meconium-stained liquor, but the child was vigorous at birth. On second day of life patient started developing fluid filled lesions on minor trauma to skin which burst spontaneously leaving behind a raw area, which subsequently healed with a scar. For the same, patient required NICU (neonatal intensive care unit) care for nearly fifteen days. A skin biopsy done at eighteen months of age was suggestive of epidermolysis bullosa dystrophica. After the diagnosis was ascertained, child was given phenytoin and promethazine for nearly three years. Over a period of time patient has developed fusion of fingers and toes (pseudo-syndactyly) with loss of nails, difficulty in mouth opening and protruding tongue. She had been immunized till five years of age and was developmentally appropriate for her age.

**Figure 1:**
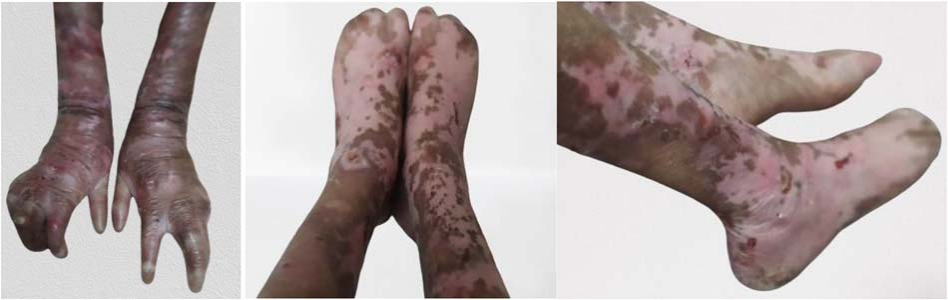
Clinical pictures of patient showing mitten-hand deformity of bilateral hands & feet.

On examination she had pallor, severe stunting and wasting [weight: 16 kg (<3^rd^ centile), height: 127 cm (<3^rd^ centile); BMI (Body Mass index):9.92 (<3^rd^ centile) as per IAP (Indian Academy of Pediatrics) growth charts], multiple superficial erosions with scabs on back, hands & legs (Fig. 1), mitten-hand deformity of bilateral hands & feet, dental caries and restricted mouth opening. Other general examination and systemic examination was unremarkable.

## Management

**Figure 2:**
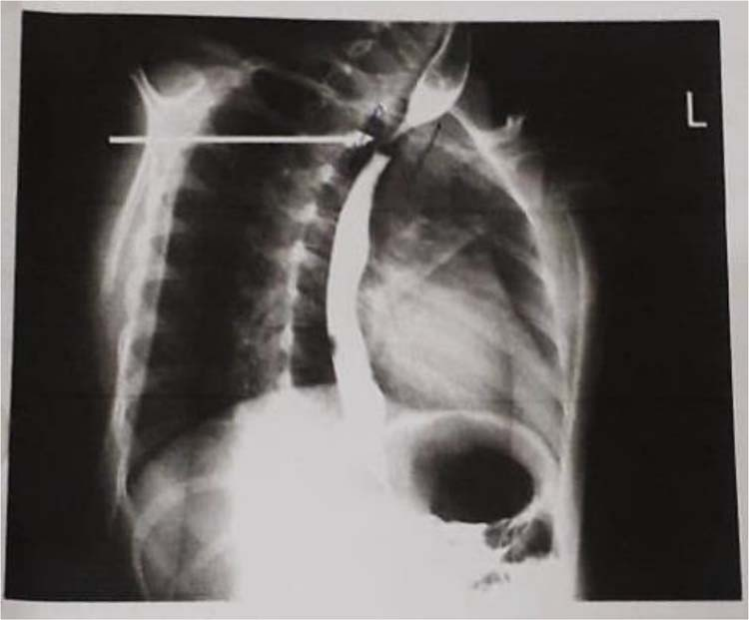
Barium swallow showing a tapered narrowing (short segment) of proximal esophagus at the level of D2 vertebral body & upstream dilatation with rest of the esophagus appearing normal in course and caliber, suggestive of either a esophageal stricture or web.

Barium swallow (Fig. 2) revealed a tapered narrowing (short segment) of proximal esophagus at the level of D2 vertebral body & upstream dilatation with rest of the esophagus appearing normal in course and caliber, suggestive of either an esophageal stricture or web. Before the patient could be taken for an endoscopy procedure, we faced many challenges owing to difficult intravenous (IV) access, restricted mouth opening, multiple dental caries and low hemoglobin (7.4 gm/dl). The anemia was probably secondary to inadequate nutrition resulting from dysphagia, as child consumed only homemade liquid foods lacking sufficient protein, calories, and nutrients. Patient required multiple tooth extraction along with blood transfusion (15 ml/kg once) before the patient could be taken for the endoscopy. Tooth extraction with widening of mouth was done under general anesthesia and fiberoptic intubation with a smaller sized endotracheal tube guided over epidural catheter at another tertiary care institute. Child had bleeding due to airway manipulation.

**Figure 3:**
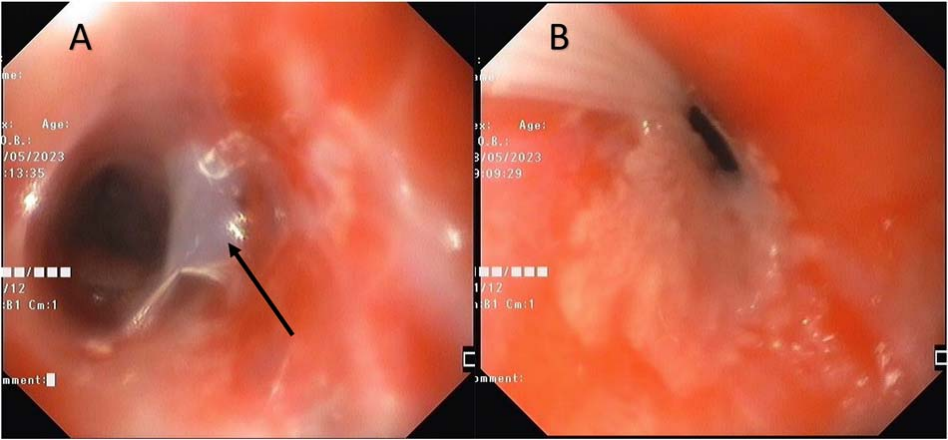
Endoscopy Images showing esophagus proximal to the stricture site. Images A and B show gelatinous mucosa (arrow mark) with easy mucosal friability.

After this procedure, the child was then planned for an endoscopic stricture dilatation. At our center patients are generally kept nil by mouth for four hours to liquids and six hours to solid food before procedure under anesthesia. Despite the difficulty in securing IV-line other alternative options like central venous line, PICC (peripherally inserted central catheter) line and intra-osseous line were avoided because of their invasive nature and to prevent infection. Patient was allowed oral rehydration solution up until two hours prior to the procedure so that IV fluids could be avoided and prevent hypoglycemia from the fasting state. Just prior to the procedure IV access was placed and any kind of sticking of the IV cannula was avoided, rather, secured by tying soft bandage around the arm. The handling of the child was restricted, and preferably, any movement the child was encouraged to do on her own. Decision was taken to avoid endo-tracheal intubation so as to minimize chances of oral and tracheal injury secondary to manipulation. All necessary equipment for securing airway were kept on stand-by. Patient was then connected to pulse oximetry probe and electrocardiography monitor for intra-procedure monitoring and to an oxygen nasal cannula at a rate of 2–4 liters per minute. She was pre-medicated with IV midazolam (0.1 mg/Kg), IV glycopyrrolate (0.004 mg/Kg) and ondansetron (0.1 mg/Kg). An initial induction dose of 2 mg/Kg of propofol and 1 mg/Kg of Ketamine was given and bolus doses of propofol were intermittently administered as required. Although mouth opening was restricted, yet, with an atraumatic mouth piece, 9.2 mm endoscope could be negotiated. A tight stricture noted in the upper esophagus at 15 cm from the incisors. Gelatinous mucosa of the esophagus (Fig. 3) with easy bruising was noted proximal to the stricture site. Using through-the-scope CRE (controlled radial expansion) fixed and wire-guided balloon dilators (Boston Scientific) stricture was serially dilated under direct visualization till 12 mm (first session till 8 mm, second session till 10 mm, third session till 12 mm) in three sessions at three-weekly intervals. Balloon was inflated till the desired pressure and pressure applied for 60 s before deflating the balloon. During the first session 20 mg of triamcinolone acetate injection was also topically applied without any mucosal invasion. Submucosal application of steroid was not attempted due to risk of perforation in an already thinned out esophageal mucosa which had a tendency to bruise easily, form blebs and heal with subsequent scarring. Topical application of steroid was not repeated as the efficacy was questionable due to very brief period of contact. Post each procedure patient spontaneously recovered from the effect of anesthetic drugs administered within 30 min to 1 h and was allowed soft semi-solid diet the same day as tolerated by the patient and patient was also given a prescription of oral antibiotics (amoxicillin-clavulanic acid at 50 mg/kg/day) for five days after every dilatation. After all the sessions of esophageal dilatation, although the patient’s dysphagia score (Mellow-Pinkas scoring) is 1, she is consuming only mashed food to avoid oral mucosal injury. There has been a 1.7 kg weight gain in a 6-month period, and she is currently not on any medications.

## Discussion

Esophageal stricture, is a debilitating complication in EB patients presenting with dysphagia, odynophagia and malnutrition. Other than esophageal stricture EB patients may experience various other esophageal issues, such as hiatal hernia, gastroesophageal reflux, esophagitis, uncoordinated peristalsis, decreased peristalsis, intramural pseudodiverticula, esophageal atony, and shortening of the esophagus [4]. Intestinal erosions when persistent also contribute to chronic protein and blood loss, leading to multifactorial anemia, hypoalbuminemia, hypoproteinemia, malabsorption, and growth retardation [3]. The various cutaneous and extra-cutaneous manifestations seen are usually seen more in patients with DEB as compared to other EB types, also evident in a recently published retrospective study of 160 children with EB [5]. DEB patients also have limited mouth opening due to scarring and contractures at the corners of the mouth. For the same, some reported cases have also required fiber-optic intubation [6]. Our patient had a diagnosis of DEB and suffered from various complications like pseudo-syndactyly, onycholysis, malnutrition, anemia and esophageal atresia along with extensive cutaneous involvement.

Many studies have reported that dysphagia complaint is present in nearly three-fourth of the EB patients and almost 70% of the patients suffer from esophageal stenosis in the first 25 years of life [7]. In a series of twenty-two EB patients with esophageal stricture undergoing stricture dilatation, the mean age of onset of dysphagia was noted to be 48 months, with the earliest occurring by 10 months of age and the average time between the onset of symptoms and dilatation was 33 months (range 1 month–10 years) [6]. This delay in diagnosis can be explained as dysphagia can also be attributed to the oropharyngeal blistering and desquamation commonly seen in severely affected DEB patients [6]. Although stricture can arise in any part of the esophagus, proximal esophagus is usually the site of stricture formation. In the same series of twenty-two patients, fourteen of these children had single strictures and of those, nine were located in the cervical esophagus. The remaining solitary strictures were located in the mid- esophagus and none were found in the distal esophagus [6]. Similarly, in other series [8–11], at least half (50%–78%) of the strictures are located in the upper third of the esophagus and middle third is more frequently affected than the distal third in the remaining of the cases.

Over the last two decades, there is a general consensus among clinicians that balloon dilatation is the preferred treatment for esophageal stricture in EB patients.

It is evident from many studies [2, 6, 11–14] that it is a safe and well-tolerated procedure. However, many of the authors prefer to do the procedure under fluoroscopic control instead of direct visualization endoscopically. The advantages stated are related to the fact that smaller biopsy channel of pediatric sized endoscopes doesn’t permit passage of a “through the scope” (TTS) balloon dilator [6]. This can be a challenge when dealing with multiple strictures, otherwise, 9.2 mm endoscope can be easily negotiated even in an infant proximal to the stricture site, which admits TTS balloon dilator and balloon dilatation can be done under direct visualization.

Other advantages of fluoroscopy guided dilatation which are stated, like, assessment of the completeness of stricture effacement and evaluation of the caliber of the balloon in relation to the caliber of the esophagus to minimize the possibility of esophageal rupture, can also be done under direct visualization without fluoroscopy assistance in the hands of an experienced endoscopist [6]. A theoretical disadvantage of the fluoroscopic method is risk of carcinoma in an already pre-disposed population secondary to repeated radiation exposure [15]. Although we used triamcinolone acetate locally, however, there isn’t strong evidence to support this. In a recently published multi-centric cohort study [15], oral corticosteroids or inhaled budesonide preparation used orally for an average of 5 days in nearly 50% of the patients, didn’t support the possible advantage of prevention of restenosis.

## Conclusion

While the endoscopic approach has less reported data [7, 15–17], it is a safe and preferred modality for stricture dilatation in EB patients when performed by an experience team. Our experience also suggests that stricture dilatation can be performed safely under total intra-venous anesthesia (TIVA) without intubating the patient. Further studies would be required to evaluate role of steroids.
